# Combining Artificial Intelligence and Human Support in Mental Health: Digital Intervention With Comparable Effectiveness to Human-Delivered Care

**DOI:** 10.2196/69351

**Published:** 2025-05-13

**Authors:** Clare E Palmer, Emily Marshall, Edward Millgate, Graham Warren, Michael Ewbank, Elisa Cooper, Samantha Lawes, Alastair Smith, Chris Hutchins-Joss, Jessica Young, Malika Bouazzaoui, Morad Margoum, Sandra Healey, Louise Marshall, Shaun Mehew, Ronan Cummins, Valentin Tablan, Ana Catarino, Andrew E Welchman, Andrew D Blackwell

**Affiliations:** 1 ieso Digital Health Ltd Cambridge United Kingdom; 2 Dorset HealthCare University NHS Foundation Poole United Kingdom

**Keywords:** mental health, anxiety, external control, synthetic control, digital intervention, smartphone, human-in-loop, AI

## Abstract

**Background:**

Escalating mental health demand exceeds existing clinical capacity, necessitating scalable digital solutions. However, engagement remains challenging. Conversational agents can enhance engagement by making digital programs more interactive and personalized, but they have not been widely adopted. This study evaluated a digital program for anxiety in comparison to external comparators. The program used an artificial intelligence (AI)–driven conversational agent to deliver clinician-written content via machine learning, with clinician oversight and user support.

**Objective:**

This study aims to evaluate the engagement, effectiveness, and safety of this structured, evidence-based digital program with human support for mild, moderate, and severe generalized anxiety. Statistical analyses sought to determine whether the program reduced anxiety more than a propensity-matched waiting control and was statistically noninferior to real-world, propensity-matched face-to-face and typed cognitive behavioral therapy (CBT).

**Methods:**

Prospective participants (N=299) were recruited from the National Health Service (NHS) or social media in the United Kingdom and given access to the digital program for up to 9 weeks (study conducted from October 2023 to May 2024). End points were collected before, during, and after the digital program, as well as at a 1-month follow-up. External comparator groups were created through propensity matching of the digital program sample with NHS Talking Therapies (NHS TT) data from ieso Digital Health (typed CBT) and Dorset HealthCare (DHC) University NHS Foundation Trust (face-to-face CBT). Superiority and noninferiority analyses were conducted to compare anxiety symptom reduction (change on the 7-item Generalized Anxiety Disorder Scale [GAD-7]) between the digital program group and the external comparator groups. The program included human support, and clinician time spent per participant was calculated.

**Results:**

Participants used the program for a median of 6 hours over 53 days, with 232 of the 299 (77.6%) engaged (ie, completing a median of 2 hours over 14 days). There was a large, clinically meaningful reduction in anxiety symptoms for the digital program group (per-protocol [PP; n=169]: mean GAD-7 change –7.4, d=1.6; intention-to-treat [ITT; n= 99]: mean GAD-7 change –5.4, d=1.1). The PP effect was statistically superior to the waiting control (d=1.3) and noninferior to the face-to-face CBT group (*P*<.001) and the typed CBT group (*P*<.001). Similarly, for the ITT sample, the digital program showed superiority to waiting control (d=0.8) and noninferiority to face-to-face CBT (*P*=.002), with noninferiority to typed CBT approaching significance (*P*=.06). Effects were sustained at the 1-month follow-up. Clinicians overseeing the digital program spent a mean of 1.6 hours (range 31-200 minutes) of clinician time in sessions per participant.

**Conclusions:**

By combining AI and human support, the digital program achieved clinical outcomes comparable to human-delivered care, while significantly reducing the required clinician time by up to 8 times compared with global care estimates. These findings highlight the potential of technology to scale evidence-based mental health care, address unmet needs, and ultimately impact quality of life and reduce the economic burden globally.

**Trial Registration:**

ISRCTN Registry ISRCTN52546704; http://www.isrctn.com/ISRCTN52546704

## Introduction

Mental health conditions are one of the economic and health care challenges of our time. Globally, 1 in 8 people live with a mental health condition [1], yet only 1 in 4 who require treatment receive it [2]. Advances in technology and widespread internet access have been pivotal in increasing access to high-quality mental health care. However, one-to-one mental health care is inherently limited in its ability to meet the rising mental health demand, and there remains a significant shortage of therapists: there are only 4 psychiatrists per 100,000 people globally [3], and 58% of the US population live within a health workforce shortage area [4]. Technology is primed to enable massive scaling of mental health interventions to increase both access and quality of support worldwide [5].

Rapid advances in technology, computing, and artificial intelligence (AI) in recent years have led to a rise in the development of digital interventions aiming to solve this scalability problem, and there are an estimated 10,000-20,000 smartphone apps available for mental health support [6,7]. These solutions have the potential to enable timely access to support when needed, negate the logistical challenges of attending regular appointments, offer greater patient choice, and reduce the burden on therapists and health care services [8]. However, real-world usage of many digital mental health solutions—most of which are self-led—has been poor [9-11]. Despite a reported willingness of patients to adopt smartphone apps [12], 1-month retention rates are typically under 6% [13]. AI-powered conversational agents improve engagement with digital mental health interventions by providing a more interactive and personalized experience compared with self-guided activities [14]. Currently, most solutions with conversational agents—that make up reportedly around 5% of digital mental health apps [15]—rely on tree-based dialogue systems with templated responses and are yet to adopt the latest in generative AI technology. This is driven by the lag between technological advancements and clinical research, along with concerns about patient safety regarding the unpredictable nature of large language model–generated output. Despite this, meta-analytic evidence indicates that having an automated, interactive dialogue system, even if rule-based, can help reduce attrition rates [16] and show promising efficacy [15,17,18]. Moreover, a recent meta-analysis of mental health apps for symptoms of anxiety and depression found a small pooled clinical effect size (*g*=0.26) and highlighted that only 48% delivered content based on cognitive behavioral therapy (CBT) principles [15], a “gold-standard” evidence-based approach for anxiety and depression [19]. Improving access is crucial, but equally vital is ensuring the support available to patients is engaging and effective.

NHS Talking Therapies (NHS TT; formerly Improving Access to Psychological Therapies) is a world-leading initiative designed to increase access to and improve the delivery of mental health treatment in the United Kingdom. Fundamental to the success of NHS TT is systematic outcomes monitoring, use of evidence-based treatment protocols, and an appropriately trained and supervised workforce [20]. The acceleration of telehealth and expansion of care delivery through digital platforms (eg, typed conversations) have also enabled insights into the relationship between the active components of evidence-based treatments and clinical outcomes [21,22]. Combining this approach with the scalable, systematic delivery of evidence-based protocols through digital tools offers the opportunity to reduce heterogeneity across the provision of mental health care worldwide, and accelerate large-scale scientific research to further enhance treatment quality and personalization [23]. High-quality, accessible digital mental health care has the potential to maximize impact globally by both improving patient quality of life and reducing the growing economic burden of mental health on health systems and society [24,25].

In this study, we evaluated a digital program that uses this approach to alleviate mild, moderate, and severe symptoms of generalized anxiety in adults. The program was designed to maximize engagement and effectiveness using (1) a structured evidence-based program drawing on principles from traditional CBT [26] including third-wave approaches, that is, acceptance and commitment therapy (ACT) [27], and (2) an AI-powered conversational agent to deliver prewritten, clinician-crafted content in a personalized way through an interactive tree-based dialogue system. The system uses advanced natural understanding models to process the natural language input by users. In addition, a dedicated human clinical and user support service was designed to wrap around the digital program, following previous research that human support significantly improves engagement with digital interventions [12,28]. This service was developed to provide appropriate support while maintaining the scalability of the digital solution.

This study aimed to measure the engagement, clinical effectiveness, acceptability, and safety of this digital program with human support. Evidence of the effectiveness of a digital intervention is often established through the comparison between the intervention and a waitlist control or self-led nondigital treatment only. However, if digital programs are to provide a scalable solution to global mental health needs, we should expect them to provide comparable effectiveness to current standards of care. In this pragmatic, prospective single-intervention arm study, we compared the digital program against propensity-matched external control data from 3 groups of real-world NHS patients: (1) a waiting control with no intervention; (2) patients receiving human-delivered face-to-face CBT; and (3) patients receiving human-delivered typed CBT. While one-to-one face-to-face therapy serves as the gold standard for comparison, one-to-one typed therapy provides a more analogous comparison to the digital program under evaluation where content is predominantly delivered through written communication with the conversational agent. This study design allowed us to evaluate the comparative clinical effectiveness of the digital program to human-delivered standard care relevant for real-world implementation.

## Methods

### Study Design

This was a pragmatic, single-intervention arm study with multiple external control groups to measure the engagement, clinical effectiveness, acceptability, and safety of a digital program to alleviate symptoms of generalized anxiety in a sample of 300 UK participants. This study was conducted by ieso Digital Health (“ieso” [29]), an outpatient service provider within NHS TT delivering one-to-one human-delivered CBT via a typed modality to treat patients with common mental health disorders. The digital program evaluated here (ie, IDH-DP2-001) was developed by ieso as part of a clinical innovation program creating new scalable digital solutions for mental health support. This was an externally controlled trial, meaning comparator arms (sometimes referred to as synthetic control arms) were generated through one-to-one propensity matching of participants with real-world patients. External propensity-matched control groups were generated to evaluate the digital program in comparison to no intervention (ie, waiting control), face-to-face CBT (gold-standard benchmark), and typed CBT. This latter group provides an important comparator as it is an example of human-delivered care that closely mirrors the written content delivery within the digital program.

The digital program was delivered via a smartphone app (iPhone and Android). Following an initial clinical assessment with a qualified clinician, eligible participants downloaded the software on their personal smartphone and completed the program in their own time and according to a defined schedule. Participants were required to complete the 6-module program within 9 weeks.

**Figure 1:**
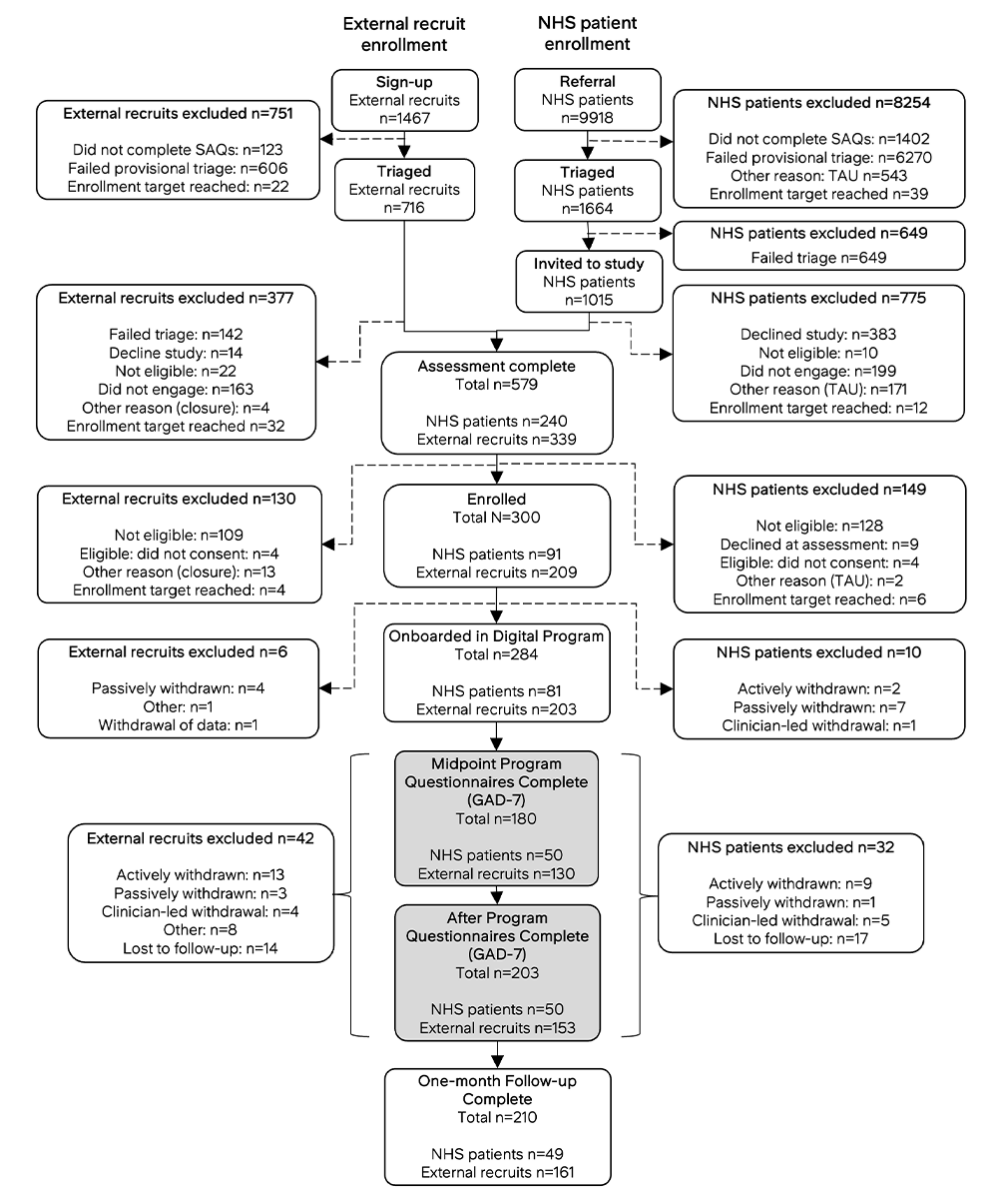
Enrollment pathways differed for external recruits (left) and patients referred to ieso for typed therapy (right), either through NHS providers or self-referral. External recruits enrolled specifically for the study via a web page, accessed through social media or email advertisements. All potential participants, regardless of recruitment source, were screened using a Self-Assessment Questionnaire (SAQ). Only patients deemed potentially eligible were invited to participate. Participants were withdrawn for various reasons: actively (upon request), passively (dropout or disengagement), clinician-led (based on clinical judgment), or other (eg, technical issues). GAD-7: 7-item Generalized Anxiety Disorder Scale; TAU: treatment as usual.

At the point of consent, all participants were asked if they were willing to participate in interviews with additional compensation offered. The subsample (based on first-come-first-served sign-up for available interview slots) attended a semistructured interview before and after the intervention to gather qualitative insights into the experience, acceptability, and perceived safety of the digital program. Findings on the acceptability of the digital program from a detailed qualitative analysis of these interviews are reported in a separate publication [30]. The trial design and participant CONSORT (Consolidated Standards of Reporting Trials) flowchart [31] are summarized in Figure 1.

### Study End Points and Data Capture

Anxiety and mood symptoms were measured before and after using the digital program, as well as at the beginning of each module within the program (a maximum of 6 symptom check-ins) using the 7-item Generalized Anxiety Disorder Scale (GAD-7) [32] and the 9-item Patient Health Questionnaire (PHQ-9) [33]. The Work and Social Adjustment Scale (WSAS) [34] and the Inflexibility Scale (30 items) of the Multidimensional Psychological Flexibility Inventory (MPFI) [35] were collected before the intervention, at the program midpoint, and after the intervention, as measures of functioning and psychological inflexibility, respectively. The following validated self-report measures were collected only at the postintervention point: the User Engagement Scale (UES) [36], the System Usability Scale (SUS) [37], and the Service-User Technology Acceptability Questionnaire (SUTAQ) [38]. A qualitative feedback survey was also administered after the intervention and at the 1-month follow-up. Demographic data were collected at enrollment and are summarized in Table 1. Findings from the SUS, UES, SUTAQ, MPFI, and feedback surveys and qualitative data from pre- and postintervention semistructured interviews are reported in a separate publication. The safety end points were serious adverse events, software deficiencies, and number of cases withdrawn based on the clinician assessment of suitability to continue with the program. Software deficiencies included malfunctions or errors of the software that could result in issues related to safety or software performance. Serious adverse events were defined as any adverse event that led to death or serious deterioration in a participant’s health.

The GAD-7 (screening only), PHQ-9 (screening only), WSAS, MPFI, SUS, SUTAQ, and demographic data were collected via ieso’s secure care delivery platform used to routinely collect patient outcomes for NHS TT. Clinical outcomes and demographic data for all control participants were also collected using this platform. GAD-7 and PHQ-9 check-ins throughout the program were collected using validated software within the smartphone app. Qualitative feedback and the UES were collected via Qualtrics (SAP SE). The safety end points were manually logged by research coordinators and clinicians following participant contact where events were reported (eg, phone calls, clinical reviews, and emails).

**Table 1:** Sample characteristics of the digital program group for both ITT^a^ and PP^b^ samples.

Demographic and category			ITT (n=299)		PP (n=169)
Age (years), mean (SD)			39.8 (12.8)		41.7 (11.8)
Baseline GAD-7^c^, mean (SD)			12.5 (3.3)		12.4 (3.4)
Baseline PHQ-9^d^, mean (SD)			8.0 (3.7)		8.0 (3.8)
**Gender, n (%)**					
	Female	240 (80.3)		137 (81.1)	
	Male	46 (15.4)		26 (15.4)	
	Other	4 (1.3)		2 (1.2)	
	Not known	9 (3.0)		4 (2.4)	
**Ethnicity, n (%)**					
	White	266 (89.0)		155 (91.7)	
	Mixed	5 (1.7)		2 (1.2)	
	Asian	14 (4.7)		6 (3.6)	
	Black/African/Caribbean/Black British	3 (1.0)		1 (0.6)	
	Other	2 (0.7)		1 (0.6)	
	Prefer not to say	9 (3.0)		4 (2.4)	
**Highest qualification, n (%)**					
	Postgraduate degree-level qualification	103 (34.4)		65 (38.5)	
	Degree-level qualification	100 (33.4)		59 (34.9)	
	Qualifications below degree level	84 (28.1)		41 (24.3)	
	No formal qualifications	2 (0.7)		1 (0.6)	
	Do not know	7 (2.3)		2 (1.2)	
	Other	1 (0.3)		0 (0)	
	Prefer not to say	2 (0.7)		1 (0.6)	
**Disability, n (%)**					
	Disability	56 (18.7)		33 (19.5)	
	No perceived disability	232 (77.6)		132 (78.1)	
	Prefer not to say	11 (3.7)		4 (2.4)	
**Chronic health condition, n (%)**					
	Yes	114 (38.1)		70 (41.4)	
	No	167 (55.9)		91 (53.8)	
	Not known	18 (6.0)		8 (4.7)	
**Religion, n (%)**					
	No religion	187 (62.5)		104 (61.5)	
	Christian	71 (23.7)		45 (26.6)	
	Buddhist	1 (0.3)		1 (0.6)	
	Hindu	5 (1.7)		3 (1.8)	
	Jewish	3 (1.0)		1 (0.6)	
	Muslim	5 (1.7)		0 (0)	
	Sikh	1 (0.3)		1 (0.6)	
	Other	11 (3.7)		7 (4.1)	
	Prefer not to say	15 (5.0)		7 (4.1)	
**Sexual orientation, n (%)**					
	Heterosexual	237 (79.3)		132 (78.1)	
	Gay/lesbian	7 (2.3)		5 (3.0)	
	Bisexual	32 (10.7)		22 (13.0)	
	Other sexual orientations not listed	7 (2.3)		2 (1.2)	
	Do not know	11 (3.7)		4 (2.4)	
	Prefer not to say	5 (1.7)		4 (2.4)	
**Employment status, n (%)**					
	Employed	241 (80.6)		144 (85.2)	
	Unemployed and actively seeking work	7 (2.3)		2 (1.2)	
	Not working and not actively seeking work	39 (13.0)		19 (11.2)	
	Prefer not to say	12 (4.0)		4 (2.4)	
**Medication status, n (%)**					
	Taking	106 (35.5)		65 (38.5)	
	Not taking	193 (64.5)		104 (61.5)	

^a^ITT: intention to treat.

^b^PP: per protocol.

^c^GAD-7: 7-item Generalized Anxiety Disorder Scale.

^d^PHQ-9: 9-item Patient Health Questionnaire.

### Description of the Digital Program

The digital program (‘ieso Digital Program’; software name: IDH-DP2-001) consisted of 6 modules that used a conversational agent to guide participants through a predefined set of activities with human clinical oversight and user support. The program was intended as a first-line intervention for people primarily presenting with anxiety symptoms. The program was designed based on cognitive behavioral principles from traditional CBT and third-wave approaches, such as ACT [39,40] (see Table S1 in Multimedia Appendix 1 for module details). All of the cognitive and behavioral processes, analogies, and examples within the program were selected for their specificity in targeting symptoms of generalized anxiety.

The 6 modules consisted of an introduction module, 3 core modules, and 2 consolidation modules. The 3 core modules each consisted of 3 sessions that followed the pattern of (1) learning, (2) activity, and (3) practice. The 2 consolidation modules consisted of 2 sessions. There were 16 sessions total. The introduction and consolidation modules consisted of sessions designed for onboarding and learning consolidation, respectively. All modules began with a symptom “check-in” consisting of the GAD-7 and PHQ-9 within the software immediately before the first session within that module. Sessions were made available on a timed schedule subject to completing the prior session.

**Figure 2:**
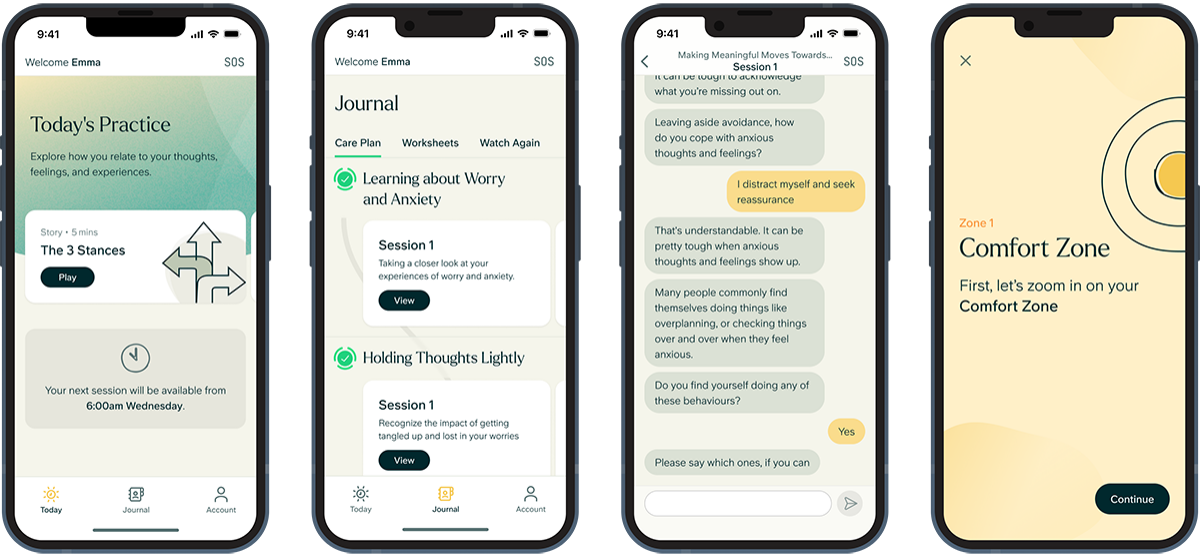
Screenshots of the ieso Digital Program user interface.

Within each session, the software used a conversational agent to guide participants through a combination of videos, educational content, conversations, and worksheets written by accredited clinicians. The software used AI models for natural language understanding, specific and tailored elements of natural language generation, and a dialogue management system. Interactions consisted primarily of text-based conversations within a tree-based dialogue system where natural language processing was used to deliver appropriate clinician-prewritten responses with controlled use of natural language generation in specific instances to enhance engagement. Partway through enrollment, with agreement from the overseeing NHS Research Ethics Committee, the software was updated to fix bugs, improve the user experience within the introductory module, and update select AI models. The final 60 participants enrolled were offered the updated software. The software version was controlled for in statistical analyses. The digital program was built in accordance with ISO 13485 [41]. Before the study, the program was registered as a UK Conformity Assessed (UKCA) marked class 1 medical device. Visuals of screens within the software are shown in Figure 2 to provide insight into the user interface that participants experienced.

### Human Support and Clinical Oversight

**Figure 3:**
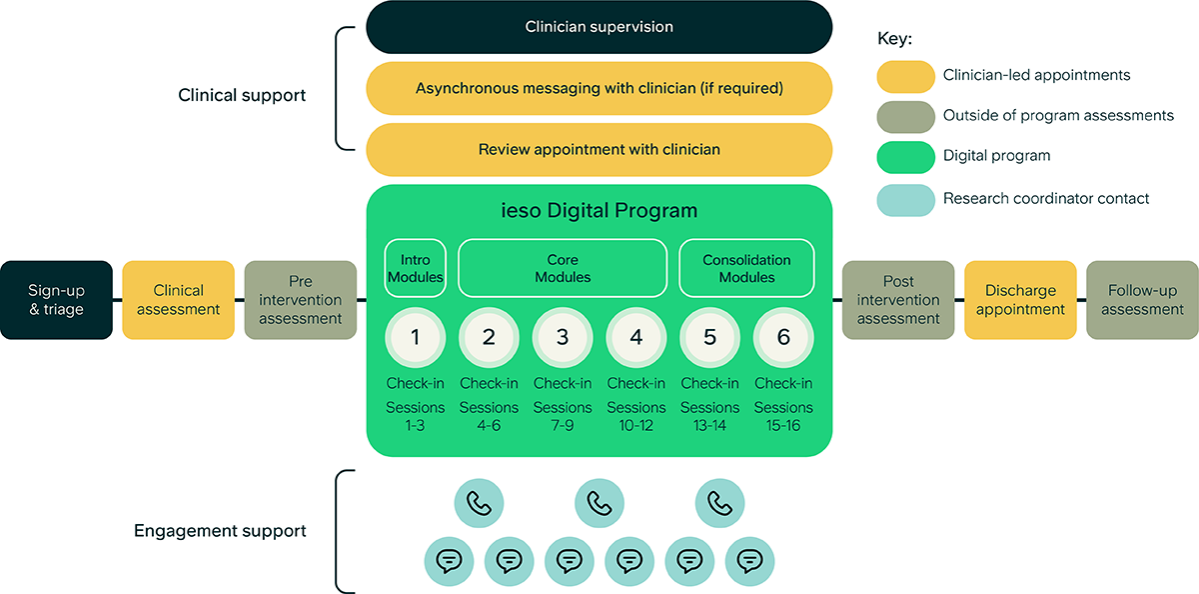
Schematic of the ieso Digital Program with human clinical and user support service and study procedures. All participants received a clinical assessment before enrollment and were offered a discharge appointment with a clinician following the program. Clinicians were available via asynchronous messaging or for a review appointment whenever needed. All participants received email or SMS text message reminders and fortnightly check-in calls throughout the program to maximize engagement delivered via the research team. The ieso Digital Program included 6 modules with a total of 16 sessions. Each module started with a symptom check-in consisting of the 7-item Generalized Anxiety Disorder Scale and 9-item Patient Health Questionnaire.

To ensure participant safety and maximize engagement and acceptability of the program, a dedicated human user and clinical support service was provided. Before enrollment, as part of the screening process, all participants received a standardized clinical assessment by a trained clinician with an accredited postgraduate qualification via typed modality. The clinician assessed the individual’s needs, determined if they were eligible for the study, and obtained informed consent. Research coordinators provided fortnightly check-in calls to all participants throughout the program and sent weekly emails or SMS text messages to remind them only if they deviated from the program schedule. The risk could be flagged through symptom monitoring of GAD-7 and PHQ-9 scores or through interaction with the research coordinators during check-in calls or ad hoc communication. The flagged risk was escalated to a clinician for review. Where appropriate, the participant would then be contacted for further risk assessment by a clinician to ensure their safety. Participants could also request an appointment with a clinician at any point to discuss their journey, particularly if they were unsure the program was working for them. At the end of the study, all participants were offered a further discharge appointment with a study clinician to discuss the next steps for their care. The support service and study procedures are illustrated in Figure 3.

### Participants

Adults with mild to severe symptoms of anxiety and a main presentation of generalized anxiety disorder (GAD) were eligible to take part. Individuals were invited to participate either following referral to ieso’s typed therapy service (either referred to ieso from the NHS Provider or via self-referral direct to ieso) or in response to online advertisements or email invitation through the NIHR (National Institute for Health Research) BioResource for Translational Research [42]. All potentially eligible individuals were assessed by a clinician in line with standardized procedures in the NHS TT manual [20] and clinicians identified a main problem descriptor with an associated International Classification of Diseases, 10th Revision (ICD-10) code [43]. Only those with a main problem descriptor of GAD were eligible. The program was not designed for individuals with a primary presenting problem of depression, and therefore, participants with a PHQ-9 score ≥16 indicative of moderate to severe symptoms of depression were signposted elsewhere for more appropriate support.

During the assessment, clinicians ensured all participants met the following eligibility criteria: (1) over the age of 18 years at the point of recruitment; (2) GAD-7 total score >7; (3) PHQ-9 total score <16; (4) primary presenting problem of GAD (based on the ICD-10 code in line with the NHS TT manual); (5) access to a smartphone and internet connection; (6) registered with a general practitioner in the United Kingdom; (7) not currently receiving psychological therapy; (8) suitable for CBT (excludes individuals with diagnosis of multiple disorders, psychotic or personality disorder, autism spectrum condition, or intellectual disability); (9) no diagnosis of an untreated mental health condition including substance misuse (except GAD or major depressive disorder); (10) did not have posttraumatic stress disorder, obsessive-compulsive disorder, or panic disorder; (11) did not have a change in psychiatric medication in the past 1 month; and (12) did not display significant risk of harm to self, to others, or from others (as established with the clinical assessment).

Any individuals who had previously participated in user research for the digital program were excluded. Participants were recruited between October 10, 2023, and February 2, 2024.

### Sample Size

Previous studies have reported attrition rates of up to 70% when measuring engagement and adherence in mental health digital programs [44-46]; therefore, we aimed to enroll 300 participants with the expectation of a 40%-70% attrition rate, resulting in a final sample of 90-180 participants. A noninferiority power analysis was conducted before the retrospective analysis of external control data to estimate the total sample size needed to quantify clinical effectiveness (ie, change in GAD-7 total score) compared with an active external control. Clinical effectiveness was defined as a change in GAD-7 score over either the course of 6 treatment sessions or until recovery was reached (if sooner than 6 sessions). A noninferiority margin of a 1.8 change in GAD-7 total score was chosen based on previous literature [47-49] (see Methods S1 in Multimedia Appendix 1 for more details). Using data from 1489 patients being treated for GAD via typed CBT, with a minimum of 6 sessions or recovery, we estimated an expected SD of GAD-7 change of 5.14. To estimate a sample size, we used the following equation (see [50]):



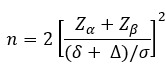



where *Z_α_* and *Z_β_* are the standard normal scores for the 1-sided significance level of 2.5% (*Z_α_*=1.96) and power of 90% (*Z_β_*=1.28), respectively; *δ* is the noninferiority level 1.8 and *σ* is the SD (5.14). A sample size of 172 was estimated for the digital program to enable a noninferiority analysis of clinical effectiveness compared with human-delivered care.

### Patient-Public Involvement

At ieso, experts-by-lived experience are involved in research and development work as members of a patient-public involvement (PPI) panel and as partners advising on ongoing work. For this study, all participant-facing documents were reviewed by members of the PPI panel. In addition, focus groups with members of the PPI panel during study conceptualization aimed to understand participant needs and expectations in the context of “keeping safe” while using the digital program, and helped develop recruitment marketing campaigns.

### External Comparator Data Source for Propensity-Matched Control Groups

External comparator data were taken from 2 NHS TT service providers: (1) ieso typed therapy data where a patient receives CBT through one-to-one communication with a qualified therapist using real-time text-based messaging and (2) Dorset HealthCare (DHC) University NHS Foundation Trust delivering face-to-face routine therapy appointments. The information captured through the data set of NHS TT is intended to support the monitoring of the implementation and effectiveness of national policy and legislation, policy development, performance analysis and benchmarking, national analysis and statistics, and national audit of NHS TT services. At registration, patients agree to the services’ terms and conditions, including the use of deidentified data for research and audit purposes, such as academic publications or conference presentations. External control data were obtained from patients referred to (1) ieso’s typed therapy service between January 2022 and December 2023, and (2) DHC between January 2017 and December 2021.

All control patients were propensity matched to enrolled participants in the digital program group based on key predictors of treatment outcomes: baseline GAD-7 scores, baseline PHQ-9 scores, age, and the presence of a chronic physical health condition (yes/no/not known) [51]. Propensity matching was conducted using the *MatchIT* package [52] in R (R Foundation) with the “nearest neighbor” methodology (average treatment effect in treated patients), matching for propensity score on a one-to-one ratio. Comparator groups showed high similarity with the digital program sample (see Table S2 in Multimedia Appendix 1). All propensity-matched control patients had a main presentation of GAD as established through the same standardized clinician assessment as the prospective participants (in line with the NHS TT manual). Treatment status and duration were matched as closely as possible and defined differently for the per-protocol (PP) and intention-to-treat (ITT) samples outlined under the “Statistical Comparison to Propensity-Matched Control Groups” section.

### Statistical Methods

#### Overview

Analyses were conducted in R [53]. A statistical analysis plan was defined before the final analyses.

#### Per-Protocol Versus Intention-to-Treat samples

The PP sample (n=169) was defined as participants who completed the minimum meaningful clinical dose (MMCD) of the program and the final postintervention GAD-7 and PHQ-9 questionnaires. This dose was defined a priori by 3 accredited cognitive behavioral therapists who evaluated the content of the program to determine the amount of content required to deliver meaningful clinical improvement on the GAD-7 scale based on their clinical experience (mean experience of 14 years delivering psychological therapy). Based on this evaluation, the MMCD was defined as completing modules 1-3 in the digital program and the module 4 check-in.

The ITT sample (n=299) included all participants who completed questionnaires at enrollment irrespective of adherence to the digital program except for 1 participant who requested that their data be deleted. Because of missing data for the preintervention WSAS, the ITT sample count for all WSAS analyses was 295.

#### Engagement and Adherence Analyses

Metrics of adherence were primarily assessed with descriptive statistics of in-software usage metrics: median and distribution of time spent in the digital program in hours; days since initialization of the program (defined based on the date that the software was downloaded); and the proportion of participants completing each session, module, and check-in. Each symptom check-in was given in the software at the start of each module. An “engaged” patient is defined as an individual who has received the minimum amount of therapy such that pre- and posttreatment measures can be collected, and clinical outcomes estimated [20]. Here we used a comparable definition of engagement based on usage of the program (including time in the program, content delivered, and number of outcomes measured), defined as completing session 1 of module 2 in the program. This is in contrast to the MMCD definition which is defined based on both usage and expected improvement in symptoms.

#### Effectiveness Analyses

Clinical effectiveness was quantified by calculating the change in anxiety symptoms, measured using the GAD-7, from baseline to final score, and estimating a within-participant effect size (Cohen *d*). A negative mean change denotes a reduction in GAD-7 total scores. Absolute Cohen *d* values are presented. The threshold for a clinically meaningful reduction in symptoms was defined as a change greater than the reliable change index of the GAD-7 scale (minimum of a 4-point reduction) [54]. A within-participant effect size for the mean change in GAD-7 scores from postintervention to 1-month follow-up was calculated to determine the short-term durability of any effects of the digital program. We also measured effectiveness by calculating the change in the PHQ-9 and WSAS scores between baseline and final scores, as well as between comparator groups. For the ITT sample, when calculating GAD-7 and PHQ-9 effectiveness, missing postintervention scores were imputed using the last observation carried forward method, such that the final score collected before disengagement or withdrawal was used.

Clinical outcomes were calculated using the following definitions: (1) improvement was defined as a reduction on the PHQ-9 or GAD-7 scales greater than or equal to the reliable change index (≥4 for the GAD-7 and ≥6 for the PHQ-9) and no reliable increase on either measure; (2) recovery was defined as a reduction on both scales to below the clinical cut-off (GAD-7 score <8 and PHQ-9 score <10); (3) reliable recovery was defined as having both improved and recovered; (4) responder rate was defined as an improvement of either ≥4 on the GAD-7 or ≥6 on the PHQ-9; and (5) remission rate was defined as having either a final GAD-7 score <8 or a final PHQ-9 score <10 for those only having started above the clinical cut-off. Definitions for improvement, recovery, and reliable recovery are equivalent to those used in NHS TT [55]. Binary clinical outcomes were compared across groups using chi-square tests. Bonferroni correction was used to account for multiple comparisons across related outcome metrics.

#### Regression Models Predicting Adherence and Effectiveness

To determine whether any demographic or study variables were associated with adherence or effectiveness, a series of regression analyses were conducted. All regression models included age, gender, highest qualification, employment status, religion, presence of a chronic physical health condition, ethnicity, reported disability, sexuality, baseline GAD-7 severity, software version, and enrollment path (referred to ieso’s typed therapy service or externally recruited) as predictors. Linear regression models were used to predict continuous dependent variables: (1) the number of sessions completed and (2) the change in GAD-7 score from baseline to final. A logistic regression model was used to predict nonadherence (ie, participants who did not complete the necessary program sessions or study assessments to be in the PP sample, with nonadherence coded as 1). Because of unequal sample sizes within demographic subcategories (eg, sexuality), groups were truncated to aid in the interpretability of findings and power of analyses.

Adherence was defined as the proportion of participants who completed each GAD-7 assessment (session) throughout their journey. For the ieso Digital Program group, each symptom check-in was at the beginning of each module within the program software (a total of 6 instances in the program). For the therapy control groups, patients completed each GAD-7 assessment as part of each attended treatment session (either face-to-face or typed) up to 6 treatment sessions. Within NHS TT, every attended treatment session includes a GAD-7 assessment. Sessions were aligned such that each symptom check-in within the digital program was associated with a treatment session for the control group. To determine whether adherence across sessions differed between groups, a generalized linear model was used to test for a session-by-group interaction.

#### Statistical Comparison to Propensity-Matched Control Groups

Three propensity-matched external control groups were created using real-world historic patient data (see the “External Comparator Data Source for Propensity-Matched Control Groups” section) to compare the clinical effectiveness of the digital program with no digital program and standard of care. For the waitlist control, only participants in the PP sample were matched (n=169) due to limited available data for matching. For the human-delivered therapy control groups, all participants were matched (n=299).

The control groups consisted of the following:

- *Waiting controls (total available sample n=576)*: patients referred for typed CBT with 2 GAD-7 scores between 4 and 10 weeks apart without having started treatment during that time (same sample used for PP and ITT analyses).
- *Therapist-delivered typed CBT (total available sample n=2210)*: patients referred for typed CBT with at least 2 scores on the GAD-7, who had completed a course of typed CBT—defined by the discharge code of “completed treatment”—and discharged with a maximum of 12 treatment sessions (PP sample), or any patient who had entered treatment, regardless of completion (ITT sample).
- *Therapist-delivered face-to-face CBT (total available sample n=753):* NHS TT patients referred to DHC who received face-to-face CBT and had a minimum of 2 and a maximum of 12 treatment sessions (PP sample), or any patient who attended treatment (ITT sample). Unlike the typed CBT comparator, due to the unavailability of discharge codes, it was not possible to use the “completed treatment” to define the PP sample for this group.

In line with the a priori–defined statistical analysis plan, a superiority analysis was conducted to test the hypothesis that the clinical effectiveness of the digital program was greater than a propensity-matched waiting control group using a between-participant *t* test (unpaired and 2-tailed). A significant *P* value (<.05) rejects the null hypothesis that there is no difference between the groups. A noninferiority analysis was conducted to test the hypothesis that the clinical effectiveness of the digital program was noninferior to the effectiveness of typed CBT or face-to-face CBT in comparison to the waiting list. The noninferiority hypothesis was that the upper confidence limit of the mean difference between groups was within the predefined noninferiority margin (<1.8). A significant *P* value (<.05) indicates the groups are noninferior. Within- and between-participant effect sizes were also estimated for the change in total score on the PHQ-9 and the WSAS to estimate the effectiveness of the digital program on low mood and work and social functioning relative to the waiting control.

### Ethical Considerations

The study was preregistered (ISRCTN ID: 52546704) and obtained ethical approval before recruitment (IRAS ID: 327897, NHS Research Ethics Committee: West of Scotland REC 4). In line with the Declaration of Helsinki, all prospective participants provided signed informed consent and were debriefed following the study. This study was conducted in accordance with Good Clinical Practice (GCP) principles. Participants were compensated for their time up to a total of £60 (US $77) in the form of vouchers based on study assessments and completion of modules within the digital program. For a subsample that participated in additional interviews, an additional £15 (US $19) voucher per semistructured interview was provided. Data from this subsample are in a separate publication.

All study data were stored in a secure environment with restricted access, and extensive quality control was conducted to ensure data integrity. ieso follows nationally and internationally recognized standards for information security (Cyber Essentials Plus, ISO 27001, and 10 National Data Guardian standards self-certified via the NHS Data Security and Protection Toolkit). All ieso study staff comply with the requirements of the UK General Data Protection Regulation (GDPR), Data Protection Act 2018, and ieso Policy with regard to the collection, storage, processing, and disclosure of personal information and will uphold the Act’s core principles.

External comparator data were taken from NHS TT service providers. The information captured through the data set of NHS TT is intended to support the monitoring of the implementation and effectiveness of national policy and legislation, policy development, performance analysis and benchmarking, national analysis and statistics, and national audit of NHS TT services. At registration, patients agree to the services’ terms and conditions, including the use of deidentified data for research and audit purposes, and academic publications or conference presentations. All NHS outcomes data were anonymized before analysis.

## Results

### Final Sample Demographics

The final sample for analysis included 299 participants of whom 240 (80.3%) were female with a mean age at baseline of 39.8 (range 18-75) years. Table 1 provides an overview of demographics and baseline symptom severity for participants in the digital program group for both the ITT and PP samples.

### Engagement and Adherence

Among the total participants, 232 (77.6%) were engaged in the program (ie, completed session 1 of module 2), involving a median of 2 hours interacting with the program content over 14 days. Of these engaged participants, 180 (77.6%) reached the MMCD (ie, completing up to check-in 4 out of 6 in the program). In the PP sample, participants completed a median of 8.7 hours over 59.6 days, and in the ITT sample, participants completed a median of 6.1 hours of program interaction over 53.1 days. The overall study attrition rate (defined as the proportion of participants who did not complete the final study questionnaires) was 96 out of 299 (32.1%). Descriptive statistics of engagement with the program are outlined in Tables 2 and 3.

**Figure 4:**
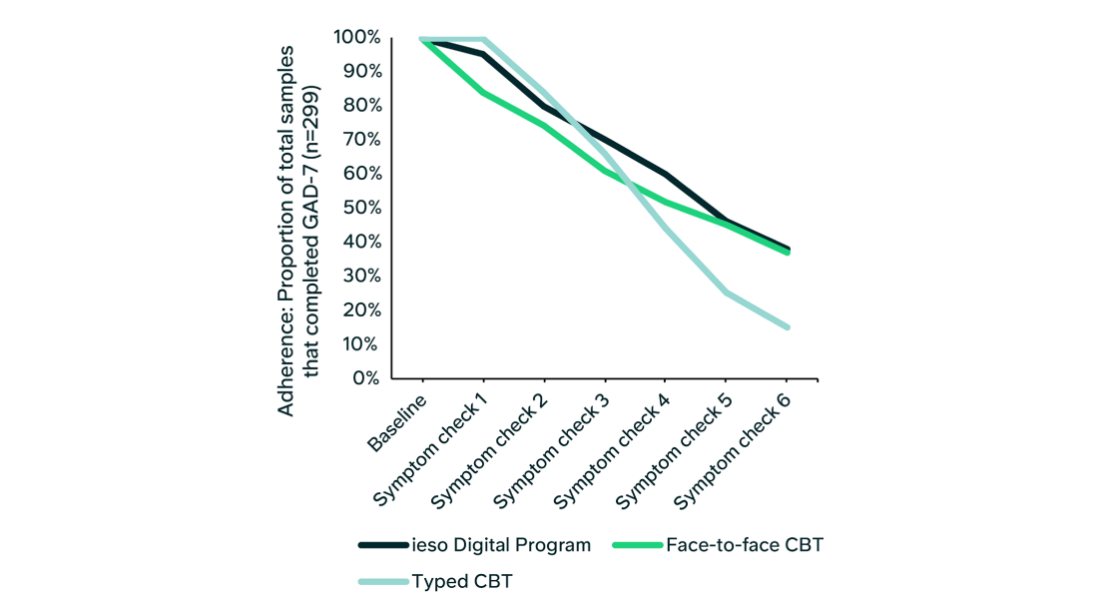
Adherence with program progression overlaid with adherence across therapy sessions for the control groups. For each group, adherence was defined based on the proportion of participants who completed each 7-item Generalized Anxiety Disorder Scale (GAD-7) assessment (symptom check) throughout their journey. The baseline was 100%, that is, all participants/patients attended a clinical assessment and had a baseline GAD-7 score. For the ieso Digital Program group, each symptom check-in was at the beginning of each module within the program software (a total of 6 instances in the program). To complete each symptom check-in within the program, participants had to finish the previous module. For the therapy control groups, patients completed each GAD-7 assessment as part of each attended treatment session (either face-to-face or typed) up to 6 treatment sessions. Within NHS Talking Therapies, every attended treatment session includes a GAD-7 assessment. Adherence rates across sessions were not significantly different between groups (see Table S3 in Multimedia Appendix 1). CBT: cognitive behavioral therapy.

To determine if adherence across sessions differed between groups, adherence rates were compared using a session-by-group interaction. There was a significant effect of session number (*b*=–10.9, SE 1.7, t_891_=–6.4, *P*<.001), but no significant session-by-group interaction for face-to-face therapy (*P*=.18) or typed therapy (*P*=.76), indicating no difference in adherence rates across groups (Figure 4; model output in Table S3 in Multimedia Appendix 1).

**Table 2:** Engagement metrics for the digital program by sample.

Metrics	n	Median time since initialization (days)	Median time interacting in the program (hours)
Engaged sample (up to module 2 session1)	232	14.0	2.0
Per-protocol sample (up to module 4 check-in)	169	59.6	8.7
Intention-to-treat sample	299	53.1	6.1

**Table 3:** Engagement metrics for the digital program by symptom check-in in the app.

Metric	n	Median time since initialization (days)	Median time interacting in the program (hours)
Module 1 check-in	284	0.0	0.03
Module 2 check-in	240	13.6	1.5
Module 3 check-in	209	23.9	2.7
Module 4 check-in	180	35.0	3.8
Module 5 check-in	138	42.9	5.0
Module 6 check-in	113	49.5	5.4

### Effectiveness

#### Anxiety Symptoms

**Figure 5:**
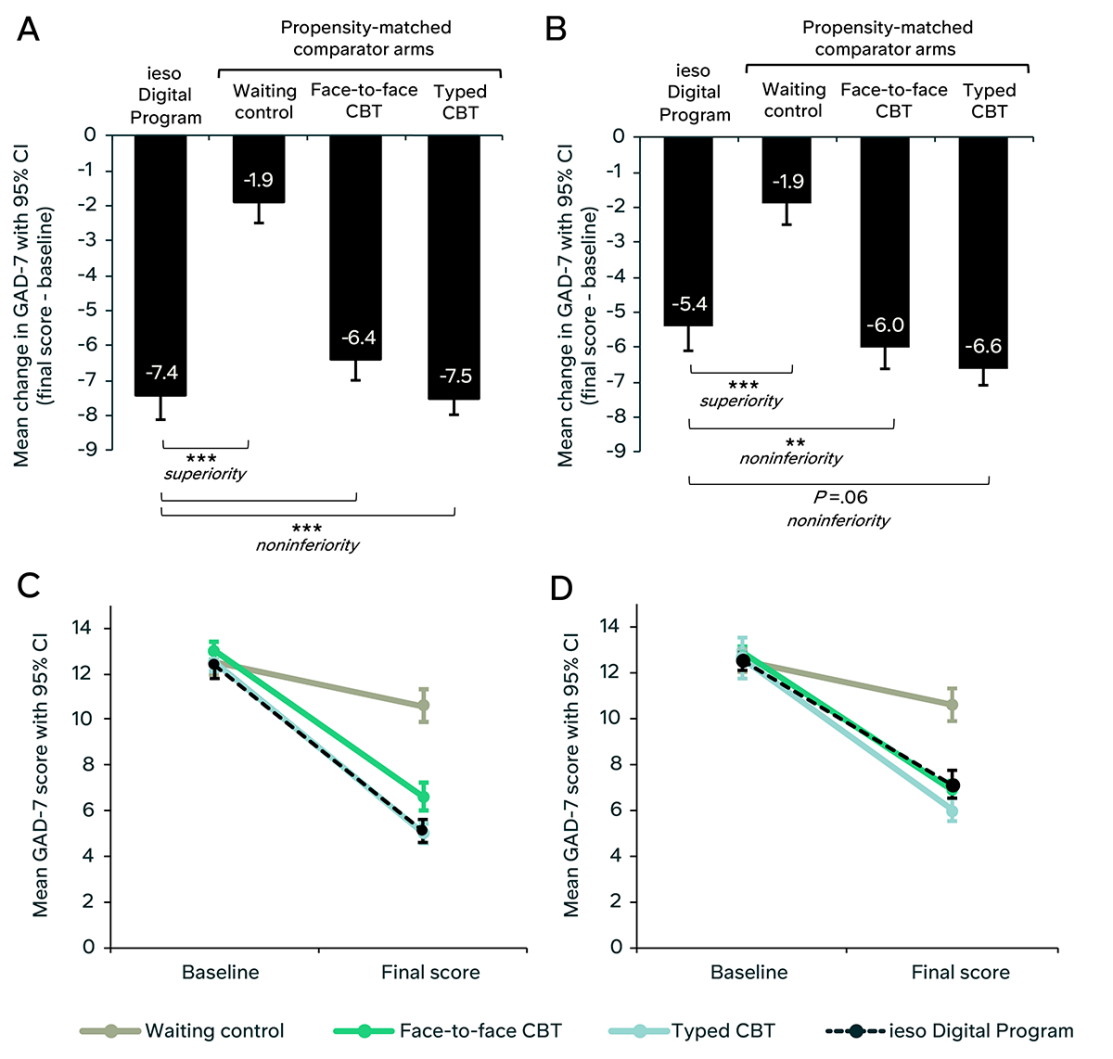
Change in anxiety symptoms from baseline to final score for the intervention sample and propensity-matched control groups. (A) Mean change (final score – baseline) in 7-item Generalized Anxiety Disorder Scale (GAD-7) scores for the per-protocol (PP) sample (n=169), propensity-matched waiting control group, face-to-face cognitive behavioral therapy (CBT) group, and typed CBT group. (B) Mean change in GAD-7 scores for the intention-to-treat (ITT) sample (n=299) and all control groups. (C) Mean GAD-7 scores at baseline and final score with 95% CIs for the PP sample (n=169) and all control groups. (D) Mean GAD-7 scores at baseline and final score with 95% CIs for the ITT sample (n=299) and all control groups. **P<.005; ***P<.001.

For the PP sample, there was a clinically meaningful reduction in anxiety symptoms from baseline to final score in the digital program group (mean GAD-7 change –7.4, 95% CI –8.1 to –6.7, *d*=1.6; Figure 5 and Table 4). This reduction was significantly greater than that observed in the waiting control group (mean GAD-7 change –1.9, 95% CI –2.5 to –1.3; *P*<.001, *d*=1.3; Table 5) and statistically noninferior to both the face-to-face therapy control (mean GAD-7 change –6.4, 95% CI –7.0 to –5.8; noninferiority effect *P*<.001) and the typed therapy control (mean GAD-7 change –7.5, 95% CI –8.0 to –7.0; noninferiority effect *P*<.001). For each comparison, the upper confidence limit of the mean between-group difference was below the noninferiority margin of 1.8. Clinical outcomes were consistently greater for the digital program compared with the waiting control, and comparable across the active control arms for the PP sample. Full outcomes are reported in Tables S6 and S7 in Multimedia Appendix 1.

**Table 4:** Within-participant change in GAD-7^a^ score from baseline to final score for the digital program sample and propensity-matched control groups.

Sample and comparator		N	Baseline score, mean (SD)	Change in GAD-7 score (final score – baseline)		
				Mean (SD)	95% CI	Within-participant effect size (*d*)
**Per-protocol sample**						
	ieso Digital Program	169	12.4 (3.4)	–7.4 (4.6)	–8.1 to –6.7	1.6
	Waiting control	169	12.5 (3.3)	–1.9 (4.0)	–2.5 to –1.3	0.5
	Face-to-face CBT^b^	253	13.0 (3.1)	–6.4 (4.8)	–7.0 to –5.8	1.3
	Typed CBT	229	12.5 (3.4)	–7.5 (4.1)	–8.0 to –7.0	1.8
**Intention-to-treat sample**						
	ieso Digital Program	299	12.5 (3.3)	–5.4 (5.1)	–6.0 to –4.8	1.1
	Waiting control	169	12.5 (3.3)	–1.9 (4.0)	–2.5 to –1.3	0.5
	Face-to-face CBT	299	12.9 (3.1)	–6.0 (4.9)	–6.6 to –5.5	1.2
	Typed CBT	299	12.6 (3.5)	–6.6 (4.6)	–7.1 to –6.1	1.4

^a^GAD-7: 7-item Generalized Anxiety Disorder Scale.

^b^CBT: cognitive behavioral therapy.

**Table 5:** Between-participant effects on GAD-7^a^ change score between the digital program and each comparator group.

Sample and comparator		Mean difference from the digital program	95% CI	*P* value	Between-participant effect size (*d*)	Statistical hypothesis tested
**Per-protocol sample**						
	Waiting control	–5.5	–6.4 to –4.5	<.001	1.3	Superiority
	Face-to-face CBT^b^	–1.0	–1.9 to –0.1	<.001	0.2	Noninferiority
	Typed CBT	0.1	–0.7 to 1.0	<.001	0	Noninferiority
**Intention-to-treat sample**						
	Waiting control	–3.5	–4.4 to –2.7	<.001	0.8	Superiority
	Face-to-face CBT	0.6	–0.2 to 1.4	.002	0.1	Noninferiority
	Typed CBT	1.2	0.4 to 2.0	.06	0.2	Noninferiority

^a^GAD-7: 7-item Generalized Anxiety Disorder Scale.

^b^CBT: cognitive behavioral therapy.

For the ITT sample, there was a clinically meaningful reduction in anxiety symptoms for the digital program group (mean GAD-7 change –5.4, 95% CI –6.0 to –4.8, *d*=1.1; Figure 5 and Table 4). This reduction was significantly greater than that observed in the waiting control group (*P*<.001, *d*=0.8; Table 5), statistically noninferior to the face-to-face therapy control (mean GAD-7 change –6.0, 95% CI –6.6 to –5.5; noninferiority effect *P*=.002), and approached significance for noninferiority compared with the typed therapy control (mean GAD-7 change –6.6, 95% CI –7.1 to –6.1; noninferiority effect *P*=.06). The upper confidence limit for the mean difference in GAD-7 change between groups was 2.0, slightly exceeding the noninferiority margin.

#### Mood Symptoms

Given the specificity of the program in targeting symptoms of generalized anxiety, a significant—though smaller—effect was observed for low mood symptoms in the PP sample, as expected (mean PHQ-9 change –3.1, 95% CI –3.8 to –2.4, *d*=0.7; *P*<.001; Table 6). This reduction was significantly greater than that observed in the waiting control group (mean PHQ-9 change –1.0, 95% CI –1.5 to –0.4, *d*=0.5; between-participant effect, *P*<.001). For the ITT sample, there was a small effect observed in the digital program group (mean PHQ-9 change –1.6, 95% CI –2.1 to –1.1, *d*=0.3), which was not significantly different from the waiting control group (*P*=.11, *d*=0.1). Despite this, the PHQ-9 remission rate—based on 116 participants above the clinical cut-off at baseline—was 78 out of 116 (67.2%) for the ITT sample (Table S4 and Table S5 in Multimedia Appendix 1).

**Table 6:** Within-participant change in PHQ-9^a^ score from baseline to final score for all groups.

Sample and comparator			N		Baseline score, mean (SD)		Change in score				
						Mean (SD)		95% CI		Within-participant effect size (*d*)	
Waiting control			169		8.4 (3.4)		–1.0 (3.6)		–1.5 to –0.4		0.3
**Per-protocol sample**											
	ieso Digital Program	169		8.0 (3.8)		–3.1 (4.5)		–3.8 to –2.4		0.7	
	Face-to-face CBT^b^	253		8.5 (3.7)		–3.0 (4.8)		–3.6 to –2.4		0.6	
	Typed CBT	229		8.1 (3.5)		–4.1 (3.9)		–4.6 to –3.6		1.1	
**Intention-to-treat sample**											
	ieso Digital Program	299		8.0 (3.7)		–1.6 (4.8)		–2.1 to –1.1		0.3	
	Face-to-face CBT	299		8.4 (3.6)		–2.7 (4.8)		–3.3 to –2.2		0.6	
	Typed CBT	299		8.1 (3.6)		–3.3 (4.2)		–3.8 to –2.9		0.8	

^a^PHQ-9: 9-item Patient Health Questionnaire.

^b^CBT: cognitive behavioral therapy.

#### Work and Social Functioning

For the PP sample, there was a significant improvement in work and social functioning, as measured by the WSAS, from baseline to final score for the digital program group (mean WSAS change –5.3, 95% CI –6.2 to –4.4, *d*=0.9; *P*<.001; Table 7). This improvement was significantly greater than that observed in the waiting control group (mean WSAS change –0.1; between-participant effect *P*<.001, *d*=1.2). Similar effects were found for the ITT sample (n=295; mean WSAS change –4.7, 95% CI –5.6 to –3.8, *d*=0.7), compared with the waiting control group (*P*<.001, *d*=0.8).

**Table 7:** Within-participant change in WSAS^a^ score from baseline to final score for all groups.

Sample and comparator			n		Baseline score, mean (SD)		Change in score				
							Mean (SD)		95% CI		Within-participant effect size (*d*)
Waiting control			153		10.6 (6.1)		–0.1 (1.3)		–0.3 to 0.1		0.1
**Per-protocol sample**											
	ieso Digital Program	169		15.3 (6.4)		–5.3 (6.2)		–6.2 to –4.4		0.9	
	Face-to-face CBT^b^	253		14.1 (7.6)		–4.3 (8.6)		–5.4 to –3.3		0.5	
	Typed CBT	223		10.8 (6.4)		–4.6 (5.5)		–5.3 to –3.8		0.8	
**Intention-to-treat sample**											
	ieso Digital Program	295		14.9 (6.6)		–4.7 (6.5)		–5.6 to –3.8		0.7	
	Face-to-face CBT	299		14.1 (7.6)		–3.9 (8.3)		–4.8 to –2.9		0.5	
	Typed CBT	291		10.8 (6.3)		–3.9 (5.7)		–4.5 to –3.2		0.7	

^a^WSAS: Work and Social Adjustment Scale.

^b^CBT: cognitive behavioral therapy.

### Stratification by GAD-7 Baseline Severity

**Figure 6:**
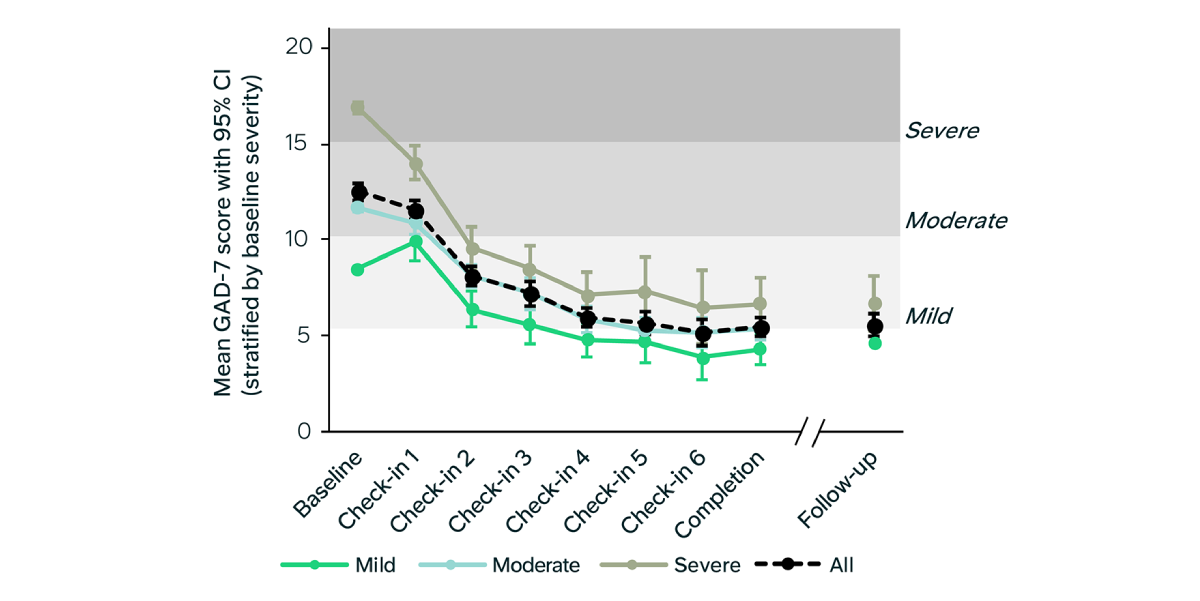
Mean reduction in anxiety symptoms across the digital program. Mean 7-item Generalized Anxiety Disorder Scale (GAD-7) score for each time point for all participants that completed the questionnaires at each time point. Trajectories split by GAD-7 baseline severity: mild, moderate, and severe (see Table S7 in Multimedia Appendix 1).

The trajectory of mean anxiety symptom reduction was steeper following the earlier program modules (Figure 6). When stratified by baseline GAD-7 severity into mild, moderate, and severe groups, the severe group showed the greatest reduction in anxiety symptoms for the PP sample (n=48; mean GAD-7 change –10.7, 95% CI –12.3 to –9.2, *d*=2.0) and the ITT sample (n=87; mean GAD-7 change –7.9, 95% CI –9.2 to –6.6, *d*=1.3; Figure 6 and Table S6 in Multimedia Appendix 1). By the end of the program, participants in both the moderate and severe baseline GAD-7 groups had mean scores that fell within the mild range. These groups also demonstrated the greatest improvements in PHQ-9 scores and showed the largest gains in work and social functioning, indicating substantial overall clinical benefit (see Table S6 in Multimedia Appendix 1).

### Durability

The mean reduction in anxiety symptoms in the digital program group was sustained at the 1-month follow-up (Figure 6). Between the final score and 1-month follow-up, there was no change in the GAD-7 mean score for the PP sample (n=166; mean change 0.0, 95% CI –0.4 to 0.5) or the ITT sample (n=210; mean change 0.0, 95% CI –0.5 to 0.4; see Table S7 in Multimedia Appendix 1). The mean reduction in low mood symptoms was also sustained. There was minimal mean change in PHQ-9 scores between postintervention and follow-up for the PP sample (mean difference 0.5, 95% CI 0.0-1.0) and the ITT sample (mean difference 0.4, 95% CI –0.1 to 0.9; see Table S8 in Multimedia Appendix 1).

### Predictors of Adherence and Effectiveness

To investigate potential drivers of program adherence, demographic and study factors were examined in relation to the number of completed sessions. Only age was significantly associated with adherence, such that older participants were more likely to complete more sessions in the program (linear regression: *F*_25,273_=1.3, *P*=.13, adjusted *R*^2^=0.03; age effect: b=0.11, SE 0.04, t_273_=2.65, *P*=.009; see Table S9 in Multimedia Appendix 1). Older participants were also more likely to be included in the PP sample (see Table S10 in Multimedia Appendix 1).

The associations between participant demographics, study factors, and change in GAD-7 score were also explored using linear regression (*F*_25,273_=3.31, *P*<.001, adjusted *R*^2^=0.16). Greater reductions in GAD-7 scores were associated with higher baseline GAD-7 scores (b=0.69, SE 0.09, t_273_=7.46, *P*<.001) and higher baseline age (b=0.08, SE 0.03, t_273_=3.0, *P*=.003; see Table S11 in Multimedia Appendix 1), such that more severe and older participants saw a larger change in GAD-7 score.

### Safety

The digital program was well tolerated, with no serious adverse events identified during the study. There was 1 report of a migraine and 2 reports of insomnia. A total of 10 software deficiencies occurred (affecting 7 participants; 9/10, 90%, occurred before the software update), primarily due to technical issues or difficulties with the conversational agent understanding users. In all instances, participants were offered an appointment to discuss any potential impact on their mental health and reminded of their right to withdraw. These instances resulted in 1 active participant withdrawal. Across the study, 10 participants were withdrawn by a study clinician following a conversation with the participant. These withdrawals were related to the study exclusion criteria and suitability for the program, rather than concerns about the safety of the digital program.

### Digital Program Clinician Time

In total, delivering the digital program required a mean of 1.6 hours (97 minutes; range 31-200 minutes) of clinician time (defined as time spent in sessions with participants) per participant. This included 299 assessments (mean 66 minutes; range 31-105 minutes), 47 review appointments (mean 32 minutes; range 14-60 minutes across 46 participants), and 173 discharge appointments (mean 44 minutes; range 13-76 minutes).

## Discussion

### Principal Findings

This study demonstrates that an evidence-based, human-supported digital program for adults with mild, moderate, and severe anxiety produced a large clinically meaningful reduction in anxiety symptoms. This was significantly greater than a propensity-matched waiting control and noninferior to real-world face-to-face and typed human-delivered CBT for those who meaningfully engaged with the program. ITT analyses also showed a significant reduction in anxiety across the digital program sample that was significantly greater than a waiting control, noninferior to face-to-face CBT, and approaching significance for noninferiority to typed CBT. Engagement with the digital program was high and participants adhered to the program at a similar rate to the external therapy control groups. The program included human clinical oversight, with clinicians spending on average 1.6 hours per participant. This study shows that a digital program for anxiety, with human support, can deliver a comparable reduction in anxiety symptoms to human-delivered care with significantly reduced clinician time. By integrating technology and human support, this study demonstrates the potential to expand global access to high-quality, effective mental health care.

The large clinical effect of the digital program across participants even with moderate or severe symptoms highlights the clinical value of the combined program content and human support. Here, the PP (*d*=1.3) and ITT (*d*=0.8) effect sizes relative to the waitlist are larger than the pooled effect size reported in a recent meta-analysis of digital interventions without any blended-care component (n comparisons=96, *g*=0.26) [15]. Unlike the PP sample, which is designed to demonstrate the clinical effectiveness of an intervention when the intervention is adhered to, the ITT sample provides an estimate of effectiveness more reflective of the real-world context by accounting for disengagement. The large ITT effect was significantly noninferior to face-to-face therapy, and approaching significance for noninferiority to typed therapy (*P*=.06). The observed difference between the digital program and typed therapy groups suggests that typed therapy was slightly more effective; however, the CI for the mean difference between groups included values both below and slightly above the noninferiority margin. Given the significant noninferiority effect for the PP sample, this suggests that further enhancements to engagement and the user experience with the program could improve real-world population effectiveness relative to standard care. Human-delivered care enables greater flexibility in responding to patient concerns and adapting content compared with a digital program. The comparable clinical effects and adherence rates across groups, particularly for the PP sample, indicates the potential of this digital program to significantly impact real-world patient outcomes.

Ensuring clinical effects are durable is highly important given the high relapse and recurrence rates that impact both patient quality of life and economic health care costs [56-58]. Incorporating cognitive and behavioral principles into daily life through practical exercises can enable meaningful behavioral change that persists beyond the treatment end. Here, both the persistent clinical effect at 1-month follow-up and the significant improvement in the impact of anxiety on participants’ day-to-day functioning (as measured with the WSAS) highlight the potential of the digital program to instigate long-lasting behavioral change. Retrospective analysis of recurrence data from electronic health records is needed to accurately measure the persistence of the clinical effect in the real world over a longer follow-up period.

The engagement rate of the digital program (232/299, 77.6%) and time to reach “engaged” (~2 hours of program interaction over 2 weeks) are comparable to engagement rates and time in therapy observed in NHS TT services for the treatment of GAD (70%; 2022-2023) [59]. Adherence rates across groups in the study were also similar. The average program interaction time (median 6.1 hours) across the ITT sample was greater than that reported for similar app-based interventions (eg, median 3.4 hours) [60], indicating high engagement with the program. Study attrition was higher than previous reports from studies of conversational agent–delivered mental health interventions (96/299, 32.1%) [16], yet similar to real-world global treatment dropout rates (~20-40%) [61,62]. This may be due to the pragmatic design of the study: 91 of the 299 (30.4%) participants recruited through ieso’s therapy referrals could choose to withdraw at any time and immediately access one-to-one human-delivered therapy; and participants had the option to discuss their progress or any issues with the clinical team at any point. These factors could have increased withdrawal rates more than previous studies, but more readily reflect real-world patient choice and clinical decision-making.

### Strengths

To our knowledge, this study is the first to compare the effectiveness of a digital program with standard of care using external propensity-matched comparator groups from real-world patient data. There is increasing acceptability for the use of externally controlled clinical trials [63-66] made possible by the availability of large-scale, standardized data sets. Generating external comparator groups reduces patient burden and study costs as well as avoids delaying treatment for the comparator group receiving no intervention [67]. Here, control groups were of high quality according to the validity criteria proposed by Thorlund and colleagues [65]: (1) control data were drawn from real-world NHS TT services, using the same clinical assessments, outcomes, and data collection procedures (in accordance with the NHS TT manual) as the prospective participants; (2) controls were selected for their highest similarity in baseline characteristics to the digital program group due to the propensity-matching procedure (see Table S2 in Multimedia Appendix 1); and (3) an a priori power analysis ensured that the sample sizes were adequate to test for noninferiority. However, creating standard-of-care control arms that are directly comparable to a novel digital program is challenging due to differences in defining comparable doses, treatment completion, and accounting for study-specific assessments. Moreover, the lack of randomization in this study means that selection bias and the effects of unmeasured variables are not controlled for. Randomization remains the gold standard for measuring efficacy in clinical trials, as it reduces bias and increases confidence that the outcomes are attributable to the intervention itself. However, effect sizes observed in randomized trials often do not generalize to real-world settings, where outcomes may be influenced by patients’ treatment preferences. This pragmatic study design may therefore more accurately reflect effectiveness in a real-world context and offers a quicker, more cost-effective method for estimating impact—ultimately reducing the time from intervention development to patient benefit.

The clinical effect and engagement rate reported in this study may have been driven by a combination of 3 key features of the digital program: (1) a curated and structured evidence-based program, (2) a conversational agent for delivering program content, and (3) a human user and clinical support model similar to standard health care delivery. First, the structured evidence-based program was curated by a team of accredited cognitive behavioral therapists with an average of 14 years of direct clinical experience. The program incorporated principles from traditional CBT [26], including third-wave approaches such as ACT. This approach encourages individuals to accept their thoughts and feelings while committing to actions that align with their values. A growing body of evidence indicates that ACT is as effective as other forms of CBT for anxiety disorders [68-70], and it has been shown to be both acceptable and engaging when delivered through a digital program for GAD [71,72].

Second, a conversational agent was used to personalize content delivery and enhance engagement. Despite the rapid growth in AI conversational agent development, the use of this technology remains rare in digital mental health interventions—currently used in only about 5% of programs [15]—though this is rapidly evolving. Most existing systems use a tree-based dialogue approach, where natural language processing analyzes user input and selects responses from a predefined set of prewritten answers. However, previous research has shown that users often find this approach frustrating, particularly when it feels as though the agent does not understand them [73,74]. Recent advances in large language model development now make it possible to flexibly generate personalized language, creating a more engaging user experience. In this study, the digital program primarily used a tree-based dialogue system, with controlled use of natural language generation in specific instances to enhance engagement. Increased use of generative technology and reduced reliance on tree-based approaches will continue to enhance the capability of conversational agents to deliver a personalized and engaging experience. However, allowing fully autonomous language generation in the context of mental health—where patient concerns can be nuanced, complex, and influenced by social and cultural factors—poses a significant risk of harm and misuse [75]. Rigorous validation of these emerging AI technologies, coupled with a phased rollout and human oversight, will be essential to ensure patient safety [76].

Finally, a “blended” design that combines human support with conversational technology has been suggested as the key to maximizing real-world engagement [16]. Previous research has identified a lack of trust, poor user-centric design, privacy concerns, low usability, and limited support during emergencies as major barriers to engagement with digital interventions [12]. To address these issues, we modeled the intervention on real-world treatment practices—incorporating user support services, clinician referrals to the program, proactive symptom monitoring, and clinician availability to support collaborative decision-making with each participant. This service created a credible and trustworthy patient experience, which we believe positively impacted outcomes. Although the study was not designed to assess the economic value of the digital program, the average clinician time spent per participant was under 2 hours—substantially lower than current global standards of care. This represents approximately 4 times less time than a typical treatment episode for GAD in the United Kingdom (approximately 8 appointments of 45-60 minutes each; NHS Digital 2021-2022 [59]), and roughly 8 times less than the global average (approximately 15 appointments; mean across reported naturalistic studies in [77]). This new model—combining an AI-driven program with clinical support—enables the current limited supply of trained therapists to reach significantly more people than traditional care models.

### Limitations

First, compensation for time may have encouraged greater adherence to the program. Second, the prospective sample had limited low-mood symptoms. In line with the study’s exclusion criteria—based on the program’s specificity for anxiety—individuals with severe depressive symptoms were not included. Nevertheless, the propensity matching across groups accounted for this, as all groups included patients with similar baseline anxiety and depression symptoms. Third, there were differences in PP sample sizes across the control groups. These differences were likely driven by the definition of PP in each context rather than actual engagement, given the similar adherence rates across groups. Defining a comparable PP sample across external control groups is challenging due to differences in dose intensity, delivery mechanisms, data collected, and response to treatment. The PP samples for the therapy control groups were based on completed episodes of care, and thus were agnostic to therapy dose—potentially including individuals who received only a few sessions but recovered quickly. Such individuals would not have been included in the digital program PP sample, which was conservatively defined based on minimum program interaction over a 9-week period. Fourth, outcomes for the propensity-matched controls were not collected concurrently with those of the prospective digital program sample, potentially introducing temporal biases. For example, the face-to-face CBT data available for analysis spanned the COVID-19 pandemic; however, only 5 matched patients in the face-to-face control group received care during 2020-2021, and their data were not outliers—making it unlikely that this impacted the findings. Propensity matching within a pragmatic design makes these findings highly relevant for real-world implementation. However, this study was not randomized and relied on patient-reported outcomes, thereby introducing risks of unmeasured confounding factors. A prospective randomized clinical trial with additional clinician-reported outcomes will be important to confirm the clinical efficacy of the digital program.

Finally, the diversity of the digital program sample was limited, with enrolled participants predominantly White, highly educated, and female. Although this sample reflects the typical profile of patients with GAD in the United Kingdom and the United States [59,78], it also mirrors existing biases in those who currently access therapy—particularly in the United Kingdom. We attempted to increase diversity in the sample by optimizing outreach strategies and using targeted marketing efforts, such as an advertising campaign in a well-known men’s sporting magazine; however, these efforts were less successful than anticipated. Needs differ across individuals, conditions, and contexts, and a deeper understanding of the barriers to research participation is required to fully address these needs—particularly among groups that have been systematically excluded from research or where mental health stigma exists. Future research will engage specialized recruitment agencies and expand to more diverse populations. Increasing access to mental health support could play a substantial role in addressing unmet needs in underserved groups. Therefore, future work will aim to demonstrate the inclusivity of this digital program and its potential to help reduce existing health inequalities.

### Conclusions

This study demonstrates that a digital program with human support, designed for adults with symptoms of generalized anxiety, can produce outcomes comparable to human-delivered CBT while significantly reducing the required clinician time. These findings highlight the potential of digital interventions to deliver high-quality, evidence-based care at scale, addressing unmet needs worldwide. As AI technologies continue to advance, generative dialogue systems that emulate creative and flexible human language are likely to become widely accessible. This increased accessibility has the potential to radically transform how individuals seek mental health support. Our responsibility is to harness these technological advances while addressing the ethical and social challenges inherent in AI. By combining the best of technology with the best of clinical care, we can increase access to effective, safe, and engaging mental health support for all. Rigorous evidence—particularly to determine the optimal blend of human and digital support for different individuals—will be essential to accelerate precision treatment, maintain scalability, maximize uptake and adherence, and successfully integrate digital interventions into health systems.

## Appendix

Multimedia Appendix 1 Supplementary methods and tables.

## Abbreviations

ACT: acceptance and commitment therapy
AI: artificial intelligence
CBT: cognitive behavioral therapy
CONSORT: Consolidated Standards of Reporting Trials
DHC: Dorset HealthCare
GAD: generalized anxiety disorder
GAD-7: 7-item Generalized Anxiety Disorder Scale
GCP: Good Clinical Practice
GDPR: General Data Protection Regulation
ICD-10: International Classification of Diseases, 10th Revision
ITT: intention to treat
MMCD: minimum meaningful clinical dose
MPFI: Multidimensional Psychological Flexibility Inventory
NHS TT: NHS Talking Therapies
NIHR: National Institute for Health Research
PHQ-9: 9-item Patient Health Questionnaire
PP: per protocol
PPI: patient-public involvement
SUS: System Usability Scale
SUTAQ: Service-User Technology Acceptability Questionnaire
UES: User Engagement Scale
UKCA: UK Conformity Assessed
WSAS: Work and Social Adjustment Scale

## Data Availability

Owing to the potential risk of patient identification, and following data privacy policies at ieso and DHC, individual-level data are not available. Aggregated data are available upon request, subject to a data-sharing agreement with ieso and DHC. Data requests should be sent to the corresponding author and will be responded to within 30 days.

## References

[1] World Health Organization (WHO) (2022). Mental Health | Key facts. WHO.

[2] Alonso Jordi, Liu Zhaorui, Evans-Lacko Sara, Sadikova Ekaterina, Sampson Nancy, Chatterji Somnath, Abdulmalik Jibril, Aguilar-Gaxiola Sergio, Al-Hamzawi Ali, Andrade Laura H, Bruffaerts Ronny, Cardoso Graça, Cia Alfredo, Florescu Silvia, de Girolamo Giovanni, Gureje Oye, Haro Josep M, He Yanling, de Jonge Peter, Karam Elie G, Kawakami Norito, Kovess-Masfety Viviane, Lee Sing, Levinson Daphna, Medina-Mora Maria Elena, Navarro-Mateu Fernando, Pennell Beth-Ellen, Piazza Marina, Posada-Villa José, Ten Have Margreet, Zarkov Zahari, Kessler Ronald C, Thornicroft Graham, WHO World Mental Health Survey Collaborators (2018). Treatment gap for anxiety disorders is global: results of the World Mental Health Surveys in 21 countries. Depress Anxiety.

[3] (2023). Data page: psychiatrists per 100,000 people. Our World in Data.

[4] (2024). Health Professional Shortage Areas.

[5] Roland J, Lawrance E, Insel T, Christensen H (2020). The Digital Mental Health Revolution: Transforming Care Through Innovation and Scale-Up.

[6] Clay R (2021). Mental health apps are gaining traction. American Psychological Association.

[7] Torous J, Roberts LW (2017). Needed innovation in digital health and smartphone applications for mental health: transparency and trust. JAMA Psychiatry.

[8] Lattie Emily G, Stiles-Shields Colleen, Graham Andrea K (2022). An overview of and recommendations for more accessible digital mental health services. Nat Rev Psychol.

[9] Borghouts Judith, Eikey Elizabeth, Mark Gloria, De Leon Cinthia, Schueller Stephen M, Schneider Margaret, Stadnick Nicole, Zheng Kai, Mukamel Dana, Sorkin Dara H (2021). Barriers to and facilitators of user engagement with digital mental health interventions: systematic review. J Med Internet Res.

[10] Ng Michelle M, Firth Joseph, Minen Mia, Torous John (2019). User engagement in mental health apps: a review of measurement, reporting, and validity. Psychiatr Serv.

[11] Michie Susan, Yardley Lucy, West Robert, Patrick Kevin, Greaves Felix (2017). Developing and evaluating digital interventions to promote behavior change in health and health care: recommendations resulting from an international workshop. J Med Internet Res.

[12] Torous John, Nicholas Jennifer, Larsen Mark E, Firth Joseph, Christensen Helen (2018). Clinical review of user engagement with mental health smartphone apps: evidence, theory and improvements. Evid Based Ment Health.

[13] Tafradzhiyski N (2025). Mobile App Retention. Business of Apps.

[14] Boucher EM, Harake NR, Ward HE, Stoeckl Sarah Elizabeth, Vargas Junielly, Minkel Jared, Parks Acacia C, Zilca Ran (2021). Artificially intelligent chatbots in digital mental health interventions: a review. Expert Rev Med Devices.

[15] Linardon Jake, Torous John, Firth Joseph, Cuijpers Pim, Messer Mariel, Fuller-Tyszkiewicz Matthew (2024). Current evidence on the efficacy of mental health smartphone apps for symptoms of depression and anxiety. A meta-analysis of 176 randomized controlled trials. World Psychiatry.

[16] Jabir Ahmad Ishqi, Lin Xiaowen, Martinengo Laura, Sharp Gemma, Theng Yin-Leng, Tudor Car Lorainne (2024). Attrition in conversational agent-delivered mental health interventions: systematic review and meta-analysis. J Med Internet Res.

[17] Fitzpatrick Kathleen Kara, Darcy Alison, Vierhile Molly (2017). Delivering cognitive behavior therapy to young adults with symptoms of depression and anxiety using a fully automated conversational agent (Woebot): a randomized controlled trial. JMIR Ment Health.

[18] Inkster Becky, Sarda Shubhankar, Subramanian Vinod (2018). An empathy-driven, conversational artificial intelligence agent (Wysa) for digital mental well-being: real-world data evaluation mixed-methods study. JMIR Mhealth Uhealth.

[19] David Daniel, Cristea Ioana, Hofmann Stefan G (2018). Why cognitive behavioral therapy is the current gold standard of psychotherapy. Front Psychiatry.

[20] (2019). NHS Talking Therapies for anxiety and depression manual. NHS England.

[21] Ewbank M P, Cummins R, Tablan V, Catarino A, Buchholz S, Blackwell A D (2021). Understanding the relationship between patient language and outcomes in internet-enabled cognitive behavioural therapy: A deep learning approach to automatic coding of session transcripts. Psychother Res.

[22] Ewbank Michael P, Cummins Ronan, Tablan Valentin, Bateup Sarah, Catarino Ana, Martin Alan J, Blackwell Andrew D (2020). Quantifying the association between psychotherapy content and clinical outcomes using deep learning. JAMA Psychiatry.

[23] Huckvale Kit, Venkatesh Svetha, Christensen Helen (2019). Toward clinical digital phenotyping: a timely opportunity to consider purpose, quality, and safety. NPJ Digit Med.

[24] Catarino A, Harper S, Malcolm R, Stainthorpe A, Warren G, Margoum M, Hooper J, Blackwell Ad, Welchman Ae (2023). Economic evaluation of 27,540 patients with mood and anxiety disorders and the importance of waiting time and clinical effectiveness in mental healthcare. Nat. Mental Health.

[25] Taylor Heather L, Menachemi Nir, Gilbert Amy, Chaudhary Jay, Blackburn Justin (2023). Economic burden associated with untreated mental illness in Indiana. JAMA Health Forum.

[26] Fenn K, Byrne M (2013). The key principles of cognitive behavioural therapy. InnovAiT: Education and inspiration for general practice.

[27] Hayes C, Strosahl D, Wilson G (2011). Acceptance and Commitment Therapy: The Process and Practice of Mindful Change, Second Edition.

[28] Gilbody Simon, Brabyn Sally, Lovell Karina, Kessler David, Devlin Thomas, Smith Lucy, Araya Ricardo, Barkham Michael, Bower Peter, Cooper Cindy, Knowles Sarah, Littlewood Elizabeth, Richards David A, Tallon Debbie, White David, Worthy Gillian, REEACT collaborative (2017). Telephone-supported computerised cognitive-behavioural therapy: REEACT-2 large-scale pragmatic randomised controlled trial. Br J Psychiatry.

[29] ieso Digital Health.

[30] Papiernik P, Dzula S, Zimanyi M (2025). Acceptability of a conversational agent-led digital program for anxiety: a mixed-methods study of patient perspectives. PsyArXiv. Preprint posted online on February 10, 2025.

[31] Schulz KF, Altman DG, Moher D, CONSORT Group (2010). CONSORT 2010 statement: updated guidelines for reporting parallel group randomised trials. BMJ.

[32] Spitzer RL, Kroenke K, Williams JBW, Lowe B (2006). A brief measure for assessing generalized anxiety disorder: the GAD-7. Arch Intern Med.

[33] Kroenke K, Spitzer RL (2002). The PHQ-9: a new depression diagnostic and severity measure. Psychiatric Annals.

[34] Mundt JC, Marks IM, Shear MK, Greist JH (2002). The Work and Social Adjustment Scale: a simple measure of impairment in functioning. Br J Psychiatry.

[35] Rolffs Jaci L, Rogge Ronald D, Wilson Kelly G (2018). Disentangling components of flexibility via the Hexaflex model: development and validation of the Multidimensional Psychological Flexibility Inventory (MPFI). Assessment.

[36] O’Brien Hl, Cairns P, Hall M (2018). A practical approach to measuring user engagement with the refined User Engagement Scale (UES) and new UES short form. International Journal of Human-Computer Studies.

[37] Brooke J (1996). SUS: a 'quick and dirty' usability scale. Usability Evaluation in Industry (1st Edition).

[38] Hirani Shashivadan P, Rixon Lorna, Beynon Michelle, Cartwright Martin, Cleanthous Sophie, Selva Abi, Sanders Caroline, Newman Stanton P, investigators WSD (2017). Quantifying beliefs regarding telehealth: development of the Whole Systems Demonstrator Service User Technology Acceptability Questionnaire. J Telemed Telecare.

[39] Berg H, Akeman E, McDermott TJ, Cosgrove Kelly T, Kirlic Namik, Clausen Ashley, Cannon Mallory, Yeh Hung-Wen, White Evan, Thompson Wesley K, Choquette Emily M, Sturycz-Taylor Cassandra A, Cochran Gabe, Ramirez Sam, Martell Christopher R, Wolitzky-Taylor Kate B, Craske Michelle G, Abelson James L, Paulus Martin P, Aupperle Robin L (2023). A randomized clinical trial of behavioral activation and exposure-based therapy for adults with generalized anxiety disorder. J Mood Anxiety Disord.

[40] Hayes SC, Follette VM, Marsha L (2004). Mindfulness and Acceptance: Expanding the Cognitive-Behavioral Tradition.

[41] (2016). Medical devices — quality management systems — requirements for regulatory purposes (ISO 13485:2016). International Organization for Standardization (ISO).

[42] NIHR BioResource. NIHR.

[43] (2019). International Statistical Classification of Diseases and Related Health Problems 10th Revision. World Health Organization (WHO).

[44] Beatty C, Malik T, Meheli S, Sinha C (2022). Evaluating the therapeutic alliance with a free-text CBT conversational agent (Wysa): a mixed-methods study. Front Digit Health.

[45] Boucher Eliane, Honomichl Ryan, Ward Haley, Powell Tyler, Stoeckl Sarah Elizabeth, Parks Acacia (2022). The effects of a digital well-being intervention on older adults: retrospective analysis of real-world user data. JMIR Aging.

[46] Cliffe Bethany, Croker Abigail, Denne Megan, Stallard Paul (2018). Supported web-based guided self-help for insomnia for young people attending child and adolescent mental health services: protocol for a feasibility assessment. JMIR Res Protoc.

[47] Robinson Emma, Titov Nickolai, Andrews Gavin, McIntyre Karen, Schwencke Genevieve, Solley Karen (2010). Internet treatment for generalized anxiety disorder: a randomized controlled trial comparing clinician vs. technician assistance. PLoS One.

[48] Titov Nickolai, Dear Blake F, Johnston Luke, Lorian Carolyn, Zou Judy, Wootton Bethany, Spence Jay, McEvoy Peter M, Rapee Ronald M (2013). Improving adherence and clinical outcomes in self-guided internet treatment for anxiety and depression: randomised controlled trial. PLoS One.

[49] Titov N, Andrews G, Robinson E, Schwencke G, Johnston L, Solley K, Choi I (2009). Clinician-assisted internet-based treatment is effective for generalized anxiety disorder: randomized controlled trial. Aust N Z J Psychiatry.

[50] Rothmann M, Wiens B, Chan I (2016). Design and Analysis of Non-Inferiority Trials.

[51] Catarino Ana, Bateup Sarah, Tablan Valentin, Innes Katherine, Freer Stephen, Richards Andy, Stott Richard, Hollon Steven D, Chamberlain Samuel Robin, Hayes Ann, Blackwell Andrew D (2018). Demographic and clinical predictors of response to internet-enabled cognitive-behavioural therapy for depression and anxiety. BJPsych Open.

[52] Ho DE, Imai K, King G, Stuart EA (2011). MatchIt: nonparametric preprocessing for parametric causal inference. Journal of Statistical Software.

[53] R Core Team (2023). R: a language and environment for statistical computing. R Foundation for Statistical Computing.

[54] Toussaint A, Husing Paul, Gumz A, Wingenfeld Katja, Harter Martin, Schramm Elisabeth, Lowe Bernd (2020). Sensitivity to change and minimal clinically important difference of the 7-item Generalized Anxiety Disorder Questionnaire (GAD-7). Journal of Affective Disorders.

[55] Clark David M (2018). Realizing the mass public benefit of evidence-based psychological therapies: the IAPT program. Annu Rev Clin Psychol.

[56] Ali Shehzad, Rhodes Laura, Moreea Omar, McMillan Dean, Gilbody Simon, Leach Chris, Lucock Mike, Lutz Wolfgang, Delgadillo Jaime (2017). How durable is the effect of low intensity CBT for depression and anxiety? Remission and relapse in a longitudinal cohort study. Behav Res Ther.

[57] Delgadillo Jaime, Rhodes Laura, Moreea Omar, McMillan Dean, Gilbody Simon, Leach Chris, Lucock Mike, Lutz Wolfgang, Ali Shehzad (2018). Relapse and recurrence of common mental health problems after low intensity cognitive behavioural therapy: the WYLOW longitudinal cohort study. Psychother Psychosom.

[58] Shallcross Amanda J, Willroth Emily C, Fisher Aaron, Dimidjian Sona, Gross James J, Visvanathan Pallavi D, Mauss Iris B (2018). Relapse/recurrence prevention in major depressive disorder: 26-month follow-up of mindfulness-based cognitive therapy versus an active control. Behav Ther.

[59] NHS Digital (2024). NHS Talking Therapies, for anxiety and depression, annual reports, 2022-23. NHS UK.

[60] Richards Derek, Enrique Angel, Eilert Nora, Franklin Matthew, Palacios Jorge, Duffy Daniel, Earley Caroline, Chapman Judith, Jell Grace, Sollesse Sarah, Timulak Ladislav (2020). A pragmatic randomized waitlist-controlled effectiveness and cost-effectiveness trial of digital interventions for depression and anxiety. NPJ Digit Med.

[61] Wells J Elisabeth, Browne Mark Oakley, Aguilar-Gaxiola Sergio, Al-Hamzawi Ali, Alonso Jordi, Angermeyer Matthias C, Bouzan Colleen, Bruffaerts Ronny, Bunting Brendan, Caldas-de-Almeida José Miguel, de Girolamo Giovanni, de Graaf Ron, Florescu Silvia, Fukao Akira, Gureje Oye, Hinkov Hristo Ruskov, Hu Chiyi, Hwang Irving, Karam Elie G, Kostyuchenko Stanislav, Kovess-Masfety Viviane, Levinson Daphna, Liu Zhaorui, Medina-Mora Maria Elena, Nizamie S Haque, Posada-Villa José, Sampson Nancy A, Stein Dan J, Viana Maria Carmen, Kessler Ronald C (2013). Drop out from out-patient mental healthcare in the World Health Organization's World Mental Health Survey initiative. Br J Psychiatry.

[62] Olfson Mark, Marcus Steven C (2010). National trends in outpatient psychotherapy. Am J Psychiatry.

[63] (2023). Considerations for the design and conduct of externally controlled trials for drug and biological products guidance for industry (draft guidance). Food and Drug Administration (FDA).

[64] National Institute for Healthcare Excellence (NICE) (2022). NICE real-world evidence framework. NICE.

[65] Thorlund K, Dron L, Park JJH, Mills EJ (2020). Synthetic and external controls in clinical trials – a primer for researchers. CLEP.

[66] Corrigan-Curay Jacqueline, Sacks Leonard, Woodcock Janet (2018). Real-world evidence and real-world data for evaluating drug safety and effectiveness. JAMA.

[67] Patterson Beth, Boyle Michael H, Kivlenieks Michelle, Van Ameringen Michael (2016). The use of waitlists as control conditions in anxiety disorders research. J Psychiatr Res.

[68] (2015). Diagnosis: mixed anxiety conditions | Treatment: acceptance and commitment therapy for mixed anxiety disorders. Society of Clinical Psychology.

[69] Han Areum, Kim Tae Hui (2022). Efficacy of internet-based acceptance and commitment therapy for depressive symptoms, anxiety, stress, psychological distress, and quality of life: systematic review and meta-analysis. J Med Internet Res.

[70] Papola Davide, Miguel Clara, Mazzaglia Mariacristina, Franco Pamela, Tedeschi Federico, Romero Sara A, Patel Anushka R, Ostuzzi Giovanni, Gastaldon Chiara, Karyotaki Eirini, Harrer Mathias, Purgato Marianna, Sijbrandij Marit, Patel Vikram, Furukawa Toshi A, Cuijpers Pim, Barbui Corrado (2024). Psychotherapies for generalized anxiety disorder in adults: a systematic review and network meta-analysis of randomized clinical trials. JAMA Psychiatry.

[71] Kelson Joshua, Rollin Audrey, Ridout Brad, Campbell Andrew (2019). Internet-delivered acceptance and commitment therapy for anxiety treatment: systematic review. J Med Internet Res.

[72] Hemmings Nicola R, Kawadler Jamie M, Whatmough Rachel, Ponzo Sonia, Rossi Alessio, Morelli Davide, Bird Geoffrey, Plans David (2021). Development and feasibility of a digital acceptance and commitment therapy-based intervention for generalized anxiety disorder: pilot acceptability study. JMIR Form Res.

[73] Coghlan S, Leins K, Sheldrick S, Cheong M, Gooding P, D'Alfonso Simon (2023). To chat or bot to chat: ethical issues with using chatbots in mental health. Digit Health.

[74] Huang YSS, Dootson P (2022). Chatbots and service failure: when does it lead to customer aggression. Journal of Retailing and Consumer Services.

[75] (2022). The role of technology in mental healthcare. Nuffield Council on Bioethics.

[76] Stade Elizabeth C, Stirman Shannon Wiltsey, Ungar Lyle H, Boland Cody L, Schwartz H Andrew, Yaden David B, Sedoc João, DeRubeis Robert J, Willer Robb, Eichstaedt Johannes C (2024). Large language models could change the future of behavioral healthcare: a proposal for responsible development and evaluation. Npj Ment Health Res.

[77] Fluckiger Christoph, Wampold Bruce E, Delgadillo Jaime, Rubel Julian, Vîslă Andreea, Lutz Wolfgang (2020). Is there an evidence-based number of sessions in outpatient psychotherapy? - A comparison of naturalistic conditions across countries. Psychother Psychosom.

[78] Terlizzi E, Villarroel M (2020). Symptoms of generalized anxiety disorder among adults: United States, 2019. Centers for Disease Control and Prevention.

