# Correction: Combining Artificial Intelligence and Human Support in Mental Health: Digital Intervention With Comparable Effectiveness to Human-Delivered Care

**DOI:** 10.2196/88640

**Published:** 2026-01-06

**Authors:** Clare E Palmer, Emily Marshall, Edward Millgate, Graham Warren, Michael Ewbank, Elisa Cooper, Samantha Lawes, Alastair Smith, Chris Hutchins-Joss, Jessica Young, Malika Bouazzaoui, Morad Margoum, Sandra Healey, Louise Marshall, Shaun Mehew, Ronan Cummins, Valentin Tablan, Ana Catarino, Andrew E Welchman, Andrew D Blackwell

**Affiliations:** 1 ieso Digital Health Ltd Cambridge United Kingdom; 2 Dorset HealthCare University NHS Foundation Poole United Kingdom

In “Combining Artificial Intelligence and Human Support in Mental Health: Digital Intervention With Comparable Effectiveness to Human-Delivered Care” [1], the authors noted one error.

Author CH-J was previously listed with no academic qualifications. His qualification has been revised to “MSc.”

The correction will appear in the online version of the paper on the JMIR Publications website, together with the publication of this correction notice. Because this was made after submission to PubMed, PubMed Central, and other full-text repositories, the corrected article has also been resubmitted to those repositories.
